# Development of a Remote Assessment and Dynamic Response Intervention for Dementia-Related Care Needs

**DOI:** 10.1093/geroni/igaa057.2135

**Published:** 2020-12-16

**Authors:** Lyndsey Miller, Christina Reynolds, Carol Whitlatch, Joel Steele, Jeffrey Kaye

**Affiliations:** 1 Oregon Health and Science University, Portland, Oregon, United States; 2 Oregon Health & Science University, Portland, Oregon, United States; 3 Benjamin Rose Institute on Aging, Cleveland, Ohio, United States; 4 Portland State University, Portland, Oregon, United States; 5 Layton Alzheimer’s Disease Center, Portland, Oregon, United States

## Abstract

Unmet dementia-related care needs are highly prevalent, and are detrimental to the care dyad’s health and well-being, safety, and ability to age in place. The goal of this study was to develop an ecologically-valid needs assessment and integrate it with aspects of the SHARE intervention to inform values-based care planning. Using digital behavioral data collected via an actigraphy watch and multimodal sensors installed in the homes of 76 older adult couples with and without dementia, we created a prototype of the objective measures informing READyR: time spent together or separate as a dyad, exits from the home, sleep habits, physical activity, daily weight, driving habits, and medication taking behavior. These digital behavioral data were then mapped onto care values (e.g. safety, avoiding burden & autonomy) to create a values-based needs assessment protocol that is tailored to the individual care dyad. Discussion will focus on future testing and applications of READyR.

